# Turning is an important marker of balance confidence and walking limitation in persons with multiple sclerosis

**DOI:** 10.1371/journal.pone.0198178

**Published:** 2018-06-07

**Authors:** Gautam Adusumilli, Samantha Lancia, Victoria A. Levasseur, Vaishak Amblee, Megan Orchard, Joanne M. Wagner, Robert T. Naismith

**Affiliations:** 1 Department of Neurology, Washington University in Saint Louis School of Medicine, St. Louis, Missouri, United States of America; 2 School of Medicine, University of Missouri, Columbia, Missouri, United States of America; 3 School of Medicine, University of Illinois, Chicago, Illinois, United States of America; 4 Department of Physical Therapy and Athletic Training, Saint Louis University, St. Louis, Missouri, United States of America; 5 Acorda Therapeutics, Ardsley, New York, United States of America; Cardiff University, UNITED KINGDOM

## Abstract

The standard functional tool for gait assessment in multiple sclerosis (MS) clinical trials has been the 25-Foot Timed Walk Test, a measure of gait speed. Straight-line gait assessment may not reflect adequately upon balance and coordination. Walking tests with turns may add additional information towards understanding gait and balance status, and be more reflective of ambulation in the community. Understanding the impact of turn parameters on patient-reported outcomes of balance and walking would help MS clinicians better formulate treatment plans for persons with gait limitations. In this study, ninety-one persons with MS (Expanded Disability Status Score; EDSS, range: 0–6.5) were enrolled in an initial cross-sectional study. Twenty-four subjects (EDSS, range:1.0–6.0) completed a follow-up visit an average of 12 months later. Spatiotemporal gait analysis was collected at both visits using APDM Opal wireless body-worn sensors while performing the Timed-Up-and-Go (TUG) and 6-Minute Walk Test (6MWT). For both cross-sectional and longitudinal data, regression analyses determined the impact on the addition of turning parameters to stride velocity (SV), in the prediction of self-reported balance confidence (Activities-Specific Balance Confidence Scale (ABC)) and walking limitation (12-item Multiple Sclerosis Walking Scale (MSWS-12)). The addition of 6MWT peak turn velocity (PTV) to 6MWT SV increased the predictive power of the 6MWT for the ABC from 20% to 33%, and increased the predictive power from 28% to 41% for the MSWS-12. TUG PTV added to TUG SV also strengthened the relationship of the TUG for the ABC from 19% to 28%, and 27% to 36% for the MSWS-12. For those with 1 year follow-up, percent change in turn number of steps (TNS%Δ) during the 6MWT added to 6MWT SV%Δ improved the modeling of ABC%Δ from 24% to 33%. 6MWT PTV%Δ added to 6MWT SV%Δ increased the predictive power of MSWS-12%Δ from 8% to 27%. Conclusively, turn parameters improved modeling of self-perceived balance confidence and walking limitations when added to the commonly utilized measure of gait speed. Tests of longer durations with multiple turns, as opposed to shorter durations with a single turn, modeled longitudinal change more accurately. Turn speed and stability should be qualitatively assessed during the clinic visit, and use of multi-faceted tests such as the TUG or 6MWT may be required to fully understand gait deterioration in persons with MS.

## Introduction

Gait impairments are well-documented in multiple sclerosis (MS). Persons with MS (PwMS) have demonstrated decreased straight-line velocity and step length, lower limb swing asymmetry, reduced maximum hip and knee extension, and an overall decrease in propulsive force during walking [1]. The Timed 25-Foot Walk (T25FW), an objective measure of straight-line gait speed, is considered one of the gold standard measures for research and clinical gait assessment in PwMS [2]. While walking speed may reflect changes in lower extremity weakness and spasticity, it may not reflect real world walking impairment. A recent study on the T25FW, Timed-Up and Go (TUG), and Two-Minute Walking Test (2MWT) reported that gait variability in MS patients with a high rate of falls was captured least well by the T25FW [3]. Falls were highly correlated to deteriorations in balance and coordination, and the TUG test, consisting of turns and other postural transitions in addition to straight walking, was more strongly associated with these changes [3]. Turns while ambulating may therefore be important to evaluate balance and coordination.

We hypothesized that turn parameters would significantly strengthen the association between clinical walking tests and self-reported measures of walking and balance, better than the association from gait speed alone. The primary objective of this study was to evaluate this hypothesis and become better informed about the value of clinical assessments which combine straight walking and turns, Timed-Up and Go (TUG) and Six-Minute Walking Test (6MWT).

## Methods

### Participant recruitment

Approval for this study was given by the Washington University Human Research Protection Office and Institutional Review Board. Written consent was obtained for each participant prior to testing. This study, conducted at the John L. Trotter MS Center at Washington University School of Medicine, included baseline assessments in 91 PwMS, with longitudinal assessments in 24 PwMS at a mean 12.2 months (range 9–17 months). The cross-sectional study was conducted on the basis of convenience sampling over a period of six months in the MS Center, and the longitudinal study occurred over a period of six months approximately one year after the cross-sectional study. The 24 PwMS in the longitudinal study were enrolled per convenience if they already had a physician appointment on the day of testing. Inclusion criteria were age ≥18 years, confirmed diagnosis by McDonald criteria, and Expanded Disability Status Scale (EDSS) 0–6.5 [4]. Excluded were those with any other medical condition that may affect ambulation: vision impairment, lower extremity orthopedic conditions limiting ambulation, chronic pain, and morbid obesity.

### Self-reported outcome measures

PwMS completed two self-report measures prior to an instrumented gait assessment, the Activities-Specific Balance Confidence Questionnaire (ABC) and 12-Item Multiple Sclerosis Walking Scale Questionnaire (MSWS-12). The ABC quantifies self-perceived confidence in balance on a scale from 0–100% through 16 questions [5]. The total score is reported from 0–100, with lower scores reflecting a lower balance confidence. The MSWS-12 quantifies self-perceived walking limitations through 12 questions, with a total score range of 12–60 [6]. The total score is transformed to a scale of 0–100, with higher scores reflecting a greater perceived impact of MS on walking. The MSWS-12 measures self-reported difficulties in standing, walking and running capacity, and speed. The ABC scale focuses specifically upon balance and unsteadiness in terms of reaching, postural transitions, and specific community scenarios (e.g. icy sidewalks, escalators, ramps, cars). Both measures have been found to be valid and reliable in characterizing their respective features [5,6].

### MS clinical disability assessment

Disability in multiple sclerosis was measured using the Expanded Disability Status Scale (EDSS) [7], a scale scored with half point steps from 0 through 10, with increasing scores denoting greater magnitudes of disability.

### Instrumented gait assessment

Spatiotemporal parameters of gait and turning were calculated during the T25FW, TUG, and 6MWT using APDM Opal wireless sensors and Mobility Lab software (APDM, Portland, OR, USA) following manufacturers guidelines [8]. The reliability and validity of the Mobility Lab System has been established in many studies [8–10]. The T25FW is a MS clinical test of walking 25 feet [2].

Participants underwent two trials of the T25FW. TUG is a gait and postural transition test: standing up from a chair, walking 7 meters to a cone, turning around a cone, walking back to the chair, and turning to sit down [11,12]. Patients were instructed by the following phrasing “Please walk as fast as you can, but as safely as you can.” The average of 3 trials of TUG were utilized. The 6MWT is a test of walking endurance and turning: walking 50 feet back and forth around two cones for six minutes [13,14].

### Data analytical approach

Step-wise regression analyses, which can determine additive properties of variables, were primarily used to achieve the objective of the study and test the hypothesis. The baseline predictive variable was one that was established in literature and could be readily obtained from straight-line gait. The secondary predictive variables added to the baseline were turn parameters that were simple to interpret and were deemed by MS clinicians in this project as having potential to obtain using more cost-effective technology than the sensors in the future. The variables were extracted at both the initial time point for the cross-sectional study and the follow-up time point for use in the longitudinal study.

For the longitudinal analysis, further processing was done to obtain percent change between the initial and follow-up time point values for each variable. Percent change specifically was chosen because of its consistent interpretation across variables.

### Cross-sectional analysis

Stride Velocity (SV), defined as Mobility Lab's measure of gait speed (meters/second from the accelerometer in the sensors), was the baseline variable for modeling the relationship between gait parameters and self-reported outcomes [15]. Average Peak Turn Velocity (PTV) (degrees/second from the gyroscope in the sensors) for TUG and 6MWT were utilized as additional variables for the turn analysis. PTV is defined by Mobility Lab as the peak (95%) angular velocity of the trunk during turning [15]. Variables were generally normal in distribution, with minimal negative skews observed for 6MWT SV (Sk = -0.63) and TUG SV (Sk = -0.16) and slight positive skews observed for 6MWT PTV (Sk = 0.39) and TUG PTV (Sk = 0.36).

Step-wise regression analyses were conducted using IBM SPSS 18.0 software to determine the additive value of PTV to SV in the correlation of patient reported outcomes, ABC and MSWS-12. The F-test of overall significance with a one-tailed alternative hypothesis was used to determine statistically significant increases in predictive power.

### Longitudinal analysis

Percent change from baseline (%Δ) was obtained for SV, PTV, and Turn Number of Steps (TNS), defined as the number of the steps per turn, for the 24 patients who participated in the longitudinal follow-up [15]. Variables were generally normal in distribution, with slight negative skews observed for the following: 6MWT SV (both time points; Sk_i_ = -0.63, Sk_f_ = -0.41), 6MWT TNS (follow-up; Sk = -0.59), TUG SV (both time points; Sk_i_ = -0.16, Sk_f_ = -0.36), and TUG TNS (initial; Sk = -0.11). Slight positive skews were observed for: 6MWT PTV (both time points; Sk_i_ = 0.39, Sk_f_ = 0.80), 6MWT TNS (initial; Sk = 0.45), TUG PTV (both time points; Sk_i_ = 0.36, Sk_f_ = 0.32), and TUG TNS (follow-up; Sk = 0.08).

Outcomes for comparison included percent change from baseline in self-reported measures (ABC%Δ, MSWS%Δ) and clinical disability change (ΔEDSS). Step-wise regression analyses were conducted for the TUG and 6MWT to determine the value of adding PTV%Δ or TNS%Δ, to SV%Δ, in the prediction of ABC%Δ and MSWS%Δ.

## Results

### Participant demographics

Demographics for the cross-sectional and longitudinal participants were notable for median age 49, mild disability by EDSS (2.5), and disease duration of 9 years as seen in Table 1.

**Table 1:** Cross-sectional and longitudinal subject demographics.

**Cross-Sectional (n = 91)**				
	**Mean**	**SD**^**a**^	**Median**	**Range/IQR**^**c**^
Age	47	11	49	R^d^: 2–69; IQR: 41–58
EDSS	2.5	0.7	2.5	R: 0–6.5; IQR: 2.0–3.5
Disease Duration (years)	11	8	9	R: 1–37; IQR: 3–14
Gender	79 females, 12 males			
MS Sub-type^b^	87 RRMS, 3 PPMS, 1 SPMS			
Ethnicity	80 White, 9 Black, 2 Asian			
MSWS-12 Score	28.4	26.0	21.4	R: 0–76; IQR: 63–95
ABC Score	76.6	20.7	81.9	R: 27–100; IQR: 5–52
6MWT Distance Walked	1405	310	1427	R: 406–2458; IQR: 1210–1611
7-m TUG Duration	17.3	4.8	16.5	R: 10.7–43.2; IQR: 13.1–18.7
**Longitudinal (n = 24)**				
	**Mean**	**SD**	**Median**	**Range**
Age	51	11	49	R: 29–70; IQR: 44–58
EDSS	2.5	0.5	2.5	R: 1.0–6.0; IQR: 2.0–3.5
Duration between Visits (months)	12.2	2.0	11.4	R: 9–17; IQR: 12–14
Gender	21 females, 3 males			
MS Sub-type	24 RRMS, 0 PPMS, 0 SPMS			
Ethnicity	22 White, 2 Black, 0 Asian			

Age (years), EDSS, disease duration (years), MSWS-12 score, ABC score, 6MWT distance walked (feet), 7-m TUG duration (seconds), gender, ethnicity, and diagnosed MS sub-type statistics for the 91 patients in the cross-sectional analysis. Age, EDSS, duration between visits, gender, ethnicity, and MS sub-type statistics for the 24 patients in the longitudinal analysis.

^a^SD = standard deviation

^b^RRMS = relapsing-remitting multiple sclerosis, PPMS = primary-progressive multiple sclerosis, SPMS = secondary-progressive multiple sclerosis

^c^IQR = interquartile range

^d^R = range

### Cross-sectional results of turn parameters

To evaluate the extent to which the ABC and MSWS-12 were measuring similar constructs, the relationship between participant responses was determined to have an R^2^ = 64% (ABC vs. MSWS-12, r = -0.80, p < 0.0001), indicating that 36% of the variability of one scale remained unaccounted when using the other scale. In comparing the overlap between straight and turning parameters, we noted that 62–72% of the information from a straight-line walking parameter was unaccounted by a turning velocity walking parameter. Pearson correlation coefficients for the predictive measures of 6MWT and TUG were moderate and positively correlated: TUG SV vs. TUG PTV (r = 0.58, p < 0.0001), and between 6MWT SV vs. 6MWT PTV (r = 0.53; p < 0.0001).

Correlations were significantly strengthened when PTV was added to SV in the prediction of ABC. Baseline R^2^ value for ABC vs. TUG SV was 19% and increased to 28% when TUG PTV was added to the stepwise regression model (p < 0.01). Similarly, baseline R^2^ value for the prediction of ABC by 6MWT SV was 20% and increased to 33% when 6MWT PTV was added to the regression model (p < 0.01).

Similar results were found for MSWS-12 as the outcome measure. Baseline R^2^ value for the prediction of MSWS-12 by TUG SV was 27%, having increased to 36% with the addition of TUG PTV (p < 0.01). MSWS-12 by 6MWT SV increased the R^2^ from 28% to 41% with the addition of 6MWT PTV (p < 0.01).

### Longitudinal results of turn parameters

Minimal clinically importance difference (MCID) in MSWS-12 is defined as a change of 7 points [16]. Minimal detectable change (MDC) for ABC has been established as 13 points [17]. MCID in EDSS scores has been reported as a change in 1.0 if EDSS 0–5.5 and a change in 0.5 if EDSS > 6.0 [18]. MCID in shorter distance walking velocity has been reported as 20% and 0.13 m/s [19,20].

**Fig 1:**
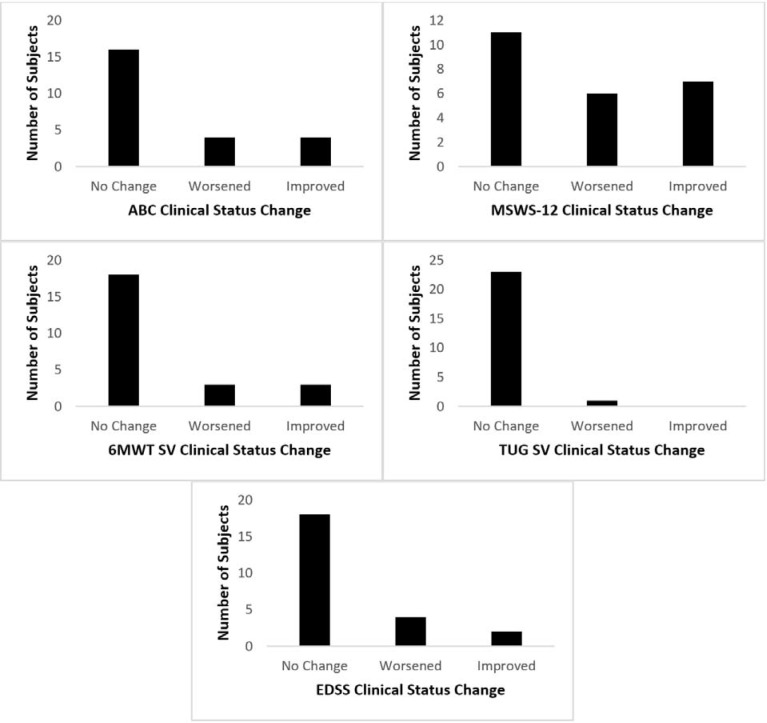
Clinical status change of ABC, MSWS-12, stride velocity in 6MWT, stride velocity in TUG, and EDSS. Longitudinal clinical status change in self-report balance confidence and walking limitation, stride velocity in a longer duration and shorter duration test, and clinical disability in 24 MS subjects.

About 1/3 of PwMS experienced a MDC balance confidence (17% improved, 17% worsened, 66% no change; Fig 1A). The majority of PwMS perceived a MCID in walking limitation (29% improved, 25% worsened, 46% no change; Fig 1B). A quarter of PwMS had a MCID in stride velocity by the 6MWT (Fig 1C), but 96% were unchanged by the TUG (Fig 1D). Most PwMS were stable in clinical disability by the EDSS (75% no change, 8% improved, 17% worsened; Fig 1E).

Two turning parameters obtained during the 6MWT improved the modeling for ABC%Δ and MSWS%Δ, but not when obtained from the TUG. Baseline R^2^ value for MSWS%Δ by 6MWT SV%Δ was 8%, improving to 27% when 6MWT PTV%Δ was added (p < 0.05). Baseline R^2^ value for ABC%Δ by 6MWT SV%Δ was 24%, improving to 33% with the addition of TNS%Δ to the regression model (p < 0.05). No improvement in modeling was observed when 6MWT PTV%Δ was added to ABC%Δ, and when 6MWT TNS%Δ was added to MSWS%Δ. For the TUG, neither PTV%Δ nor TNS%Δ improved the models for ABC%Δ and MSWS%Δ (all p > 0.05). However, the change in total percent change in TUG duration did predict R^2^ = 25% of the changes in MSWS-12 and R^2^ = 18% of the ABC.

## Discussion

This study demonstrated that turns are an important predictor of patient-reported balance confidence and walking limitation, as measuring turns improves the correlation between gait-based outcome measures and patient report of walking and balance limitations. While stride velocity parameters did correlate with the patient report of home and community ambulation, the clinical relationship was enhanced with the turning component. Consistent results were found in the cross-sectional analysis, whether using a test consisting of a single turn (TUG) or multiple turns (6MWT). For the longitudinal analysis, multiple turns appeared to more accurately capture the impairment for the 6MWT over the TUG. Although the longitudinal cohort was smaller than the cross-sectional cohort, this preliminary result suggests that including several turns could be a more sensitive way to better capture changes in balance confidence and walking limitation.

Our results also emphasize that turn speed and stability are important to assess clinically. While the sensors in the present study may not be widely available, the result may still be translated into clinical practice with the use of other cost-effective technologies, as determined by future studies. Health care professionals who want to properly assess gait should utilize a clear hallway at least 20 feet long, and ask the patient to make several turns under observation coupled with data collection from these technologies. Having the PwMS walk in a small exam room is not sufficient for proper assessment of gait that replicates day-to-day activity in the community. Interim, as studies determine the technologies most appropriate for this purpose, an experienced clinician may be able to qualitatively assess turning hesitation and number of steps by eye, as part of the comprehensive evaluation.

While the role and practicality of gait sensors in routine clinical practice remains unknown, gait sensors and quantitative measures of turning may have potential as an outcome measure for clinical trials. Current clinical measures like stride velocity during a short-distance test do not capture all components of ambulation such as fatigue, balance, and symmetry and hence may correlate poorly with PROs [21]. This may be problematic as a clinician attempts to understand how patients are feeling on a day-to-day basis in their community rather than an artificial clinic setting. Evaluating new parameters like turns during clinical assessment can help improve correlation to PROs, detect clinical changes in balance and coordination not captured by current tests, and ultimately aid a clinician in formulating the appropriate care plan.

An interesting finding from the longitudinal analysis was that different turn parameters improved predictive power of different PROs. We believe changes in TNS better corresponded to changes in ABC because wider turns with a greater number of steps from the initial testing indicate a decrease in balance confidence and more caution upon changing direction. PTV%Δ may have better predicted MSWS%Δ because decreased turn velocity suggests a decreased ability to adjust gait and change direction. Because correlation of longitudinal changes in balance confidence and walking limitation seemed to improve with a test of longer duration, this may justify the inclusion of the 6MWT or 2MWT in future therapeutic trials and other interventions.

Collectively, our results suggest that while T25FW does provide a quick and simple method for functional gait metrics, its singular measure of stride velocity can be suboptimal for clinical gait assessment in PwMS. Previous work has shown that the T25FW predicts only 18% of gait variability in patients with a high fall risk, compared to 29% for the TUG [3]. We obtained a similar result by demonstrating that TUG duration outperformed the straight-line walking component of gait (SV) at predicting longitudinal changes in walking limitation. Because the TUG also has a turn component to contribute additional predictive power, there may be merit in clinics shifting away from the T25FW to the TUG.

A number of previous studies have already suggested that MS clinics should begin using the TUG during clinical gait assessment due to its holistic nature [3,22]. One study reported the strong convergent validity the TUG has with other established measures of ambulation, such as T25FW, 6MWT, MSWS-12, and EDSS [22]. Another study demonstrated the reliability and utility of inertial sensors in quantifying the TUG [23]. With these two findings as a premise, we were able to quantitate the rationale for the added value. It is notable, however, that over 50% of the variance of PROs remains unpredicted. It may be difficult to predict this variance with tests in a closed clinic setting, as variables such as uneven lighting, incline, obstacles, pedestrians, etc often present in a community setting cannot be replicated.

Several study limitations are worth noting. Our longitudinal analysis consisted of only 24 RRMS patients, and would benefit from a future study with a larger cohort representative of the spectrum of MS. Longer follow-up and additional visits would also be recommended. While the total TUG duration and 6MWT could be used in clinic at present, and TNS could be documented in the record, newer and more affordable methods to assess turns may become available as technology advances.

Future studies would include a larger cohort, a multi-year assessment, and further broaden inclusion across the spectrum of MS subtypes and disabilities. We would like to better assess the impact and sensitivity of longitudinal analyses for TUG, 2MWT, and 6MWT. With a larger population, a stratified analysis based upon disability may show that certain tests perform optimally for specific EDSS scores. Lastly, other technology such as a motion capture system, gait mat, Fit-Bit, and smart phone app utilizing a smart phone’s in-built sensors should be compared against the APDM Opal sensors during these tests [24,25]. Because many MS clinics may be unable to afford the Opal APDM wireless sensors, finding a more accessible technology than the sensors with comparable efficiency, validity, and reliability would be ideal.

## Conclusions

Turns are an important predictor of patient-reported balance confidence and walking limitation. Although clinical disability is indeed better represented by a longer duration walking test, the TUG contains more information than the current gold-standard 25-Foot Walk Test and is of similar testing duration, thus making it a practical alternative for clinics. Further work must be done longitudinally on the TUG, 2MWT, and 6MWT, and more cost-effective technology to quantify turns must be found. In the interim, obtaining TUG duration as an outcome variable, and documenting TNS during gait assessment would be of the most value for MS clinics.

## Supporting information

S1 File Inertial sensor study data–master file. (XLSX)

S2 File Patient identifier and non-inertial sensor study data. (XLSX)
